# The new instruction librarian: a workbook for trainers and learners

**DOI:** 10.29173/jchla29931

**Published:** 2026-04-01

**Authors:** Lindsay Adoranti

**Affiliations:** Clinical Librarian, Saskatchewan Health Authority, Regina, SK, Canada

Benjes-Small C, Waltz RM. **The new instruction librarian: a workbook for trainers and learners**. 2nd ed. Chicago, IL: ALA Editions; 2026. Softcover: 256 p. ISBN: 979-8-89255-325-4. Price: USD$69.99. Available from: https://alastore.ala.org/tnil2.

Teaching in academic libraries is a multifaceted portfolio, and a vital service provided to a diversity of stakeholders including students, faculty, campus groups, and even community partners. In *The new instruction librarian: a workbook for trainers and learners*, authors Candice Benjes-Small and Rebecca Miller Waltz overview the several different roles or “hats” [1] that instruction librarians adopt.

The authors are seasoned librarians based in the United States, having held previous positions in different university and college libraries. Candice Benjes-Small is currently the head of research services at William & Mary in Virginia, serves on various library committees, and has published several articles on information literacy. Rebecca Miller Waltz works at Penn State University Libraries where she is the associate dean for learning and engagement. Rebecca is active in many library organizations and publishes on teaching and learning topics.

The book is divided into four different sections. Part one introduces the topic of instruction librarianship, explores what it means to be a teacher as a librarian in higher education, defines information literacy (IL), and outlines how conversations on IL have changed throughout library history. Part two makes up most of the book, totaling 11 chapters. Each chapter is dedicated to a different role that an instruction librarian takes on throughout their career. These roles, according to the authors, include the colleague, instructional designer, teacher, teaching partner, advocate, project manager, coordinator, and learner. Part three discusses library instruction, observation, and burnout. Finally, part four includes sample lesson plans, a lesson script, assignments, and an observation form which are referenced throughout the book.

*The new instruction librarian* is well written and easy to follow. Each chapter can be read as a standalone chapter, depending on the context of the reader. The authors list different intended audiences: recent library and information science graduates, seasoned librarians who are new to an instruction role or responsibilities, instruction librarians in a new library setting, and those who are training others in instruction. From my perspective, the book is best suited towards recent graduates and new instruction librarians because it gives general guidance, tips, advice, and introduces IL and teaching theories. Early career librarians, especially those who have not taken an IL or teaching course in library school, would find the book most useful in having an understanding in instructional theory, teaching best practices, and examples from the authors’ lived experiences.

Early career librarians would benefit from the practical tips, guidelines, and on the job stories. The theories and approaches discussed in the book such as instructional design models, information literacy, universal design for learning (UDL), and critical pedagogy provide the foundation for the tips and teaching scenarios detailed. To illustrate, tips include how to create a lesson plan, active learning exercises, classroom management tips, how to conduct learning assessments, developing a project plan, prioritizing instruction content, and using library statistics to improve and inform instruction practices.

At the end of each chapter, there is an “Ask the Experts” section which provides a fictional scenario that is associated with the “hat” [1] of the chapter. These scenarios are common issues that new instruction librarians face on the job. Two librarians (department heads, library leaders, or seasoned librarians) give their opinion on how to navigate or approach the situation. It should be noted that each response was from a professional based in the United States. It would have been beneficial if the authors included expert opinions from library workers in different countries to highlight diverse voices and lived experiences around the world.

Following the “Ask an Experts” section, there are three or four activities that readers can do to take what they read and put it into action. These include a personal reflection opportunity, a conversation starter with a supervisor or colleague, or another hands-on activity engaging with the material. Activities vary in depth which makes it easy for the reader to select one based on time and context.

Readers who do not fall into the categories of recent graduate or new instruction librarian can still find value in the book with certain chapters. For example, the chapters on project management (chapter 9) and instruction coordinator (chapter 10) would be useful for librarians who have experience in teaching and learning and are now taking on a large team project or have been promoted to a coordinator or supervisor position. An early career librarian will find these chapters to be more useful later in their career when they can apply the tips and theories to their real work context and projects, but having the knowledge is still useful.

This book is well researched and provides supplementary literature at the end of each chapter. The authors encourage readers to reflect on and develop their own opinions on teaching approaches and to expand their knowledge by seeking out the referenced material. Readers seeking supplementary readings in teaching and learning scholarship would find an abundance of useful literature referenced in this book.

The authors’ personal shared stories were a highlight of the book. Some on the job experiences shared in the text included how they solved a problem, pivoted when a plan did not go as expected, communicated with faculty, managers, or campus partners, and how they managed when a project fell through. These real experiences drew me in as a reader and made the content genuine and relatable.

A gap in this book is the majority American perspective. The authors could consider in a future edition to include diverse voices in the “Ask the Experts” section. The authors touch upon UDL, diversity, equity, and inclusion (DEI), and acknowledge how one’s own identity can influence experiences and abilities; however, going more in-depth into integrating UDL and DEI practices and approaches in library instruction would be useful.

In conclusion, *The new instruction librarian* is a well written and researched book that is best suited to readers who are recent graduates, early career instruction librarians, or seasoned librarians who are new to taking on an instruction role. Readers finding themselves in other positions may still benefit from the abundance of references provided at the end of each chapter and the chapters on project management and the library coordinator role. The strengths of this book include the practical teaching strategies, on the job stories, and mini case studies with expert advice that are informed by teaching theories. Ultimately like instruction librarianship itself, the book is multifaceted and can appeal to different readers hoping to walk away from it with practical tips, project ideas, strategies for working with campus partners, teaching best practices, reflection prompts, and much more.

## References

[1] Benjes-Small C, Waltz RM. The new instruction librarian: a workbook for trainers and learners. 2nd ed. Chicago, IL: ALA Editions; 2026. p. 33.

